# Case Report: A Case Report of Neurosyphilis Mimicking Limbic Encephalitis

**DOI:** 10.3389/fneur.2022.862175

**Published:** 2022-05-12

**Authors:** Haibing Liao, Yajing Zhang, Wei Yue

**Affiliations:** Department of Neurology, Tianjin Huanhu Hospital, Tianjin, China

**Keywords:** neurosyphilis, encephalitis, leucine-rich glioma inactivated 1 protein, penicillin, case report

## Abstract

Neurosyphilis (NS) is an infection of the central nervous system caused by Treponema pallidum. It mimics various neurological and psychiatric diseases. In recent years, there have been several NS cases that manifest as limbic encephalitis (LE). Therefore, the diagnosis of neurosyphilis in the early stages is difficult. Here, we present a case of an NS patient who presented with LE manifestation. The 62-year-old woman presented with acute clinical manifestations of gibberish speech, poor memory, and seizures. Brain MRI showed abnormal signals on the right medial temporal lobe. In addition, the patient had a positive serum leucine-rich glioma inactivated 1 (LGI1) antibody with a titer of 1:16. Therefore, an initial diagnosis of anti-LGI1 encephalitis was made. However, further tests carried out showed positive rapid plasma reagin (RPR), and treponema pallidum particle agglutination (TPPA) tests both in the serum and the cerebrospinal fluid (CSF). Therefore, uncertainty arose as to whether the patient had both anti-LGI1 encephalitis and NS or whether the LGI1 antibody and LE manifestations were due to the NS. The patient was initiated on the recommended dose of penicillin G sodium. Following treatment, the patient reported a significant improvement in clinical symptoms, normal signals in the right temporal lobe, and a negative serum LGI1 antibody. These findings suggested that NS induced the LE manifestations and the production of the LGI1 antibody. This case demonstrates that testing syphilis in patients with LE is important and positive autoimmune encephalitis (AE) antibodies in NS patients need to be viewed and interpreted with greater caution.

## Introduction

Syphilis is a sexually transmitted disease caused by the bacterium *Treponema pallidum*. It develops in four different stages: early syphilis (primary, secondary, and early latent syphilis) and late syphilis (latent and tertiary syphilis). Neurosyphilis (NS) is an infection of the central nervous system (CNS), which may occur at any stage of the infection (1). Syphilis outbreaks have been reported in some countries despite a fall in the reported incidence (2). The clinical manifestations of NS have had a dramatic change over the past 30 years. Compared with the pre-penicillin era, there has been a decline in cases of general paresis and tabes dorsalis. However, atypical forms (epilepsy, eye symptoms, stroke, confusion, or personality changes) have increased (3, 4). In recent years, several NS cases which present as limbic encephalitis (LE) have been reported, thus making it more difficult to diagnose NS in the early stage(5). Here, we report a case of a patient with NS presenting with LE manifestation. Therefore, we suggest that patients with LE manifestations should be routinely tested for syphilis to avoid misdiagnosis of NS.

## Case Presentation

A 62-year-old woman was admitted to the neurology department of Tianjin Huanhu Hospital. The patient complained of paroxysmal falls accompanied by impaired consciousness for the last nine days and paroxysmal limb twitch accompanied by gibberish speech for four days. Initially, the patient experienced tonic-clonic seizures, accompanied by impaired consciousness. Later, the patient experienced tonic seizures with head turning to the right and retained consciousness similar to faciobrachial dystonic seizures (FBDS) (Supplementary Materials S1). These incidences occurred more than ten times in a day. The patient was first seen in another hospital, and a brain MRI was done. The MRI showed no abnormalities. The patient was started on sodium valproate, diazepam, and levetiracetam (drug doses unknown). Due to no clinical improvement, the patient was referred for further management. The patient had no previous history of hypertension, coronary heart disease, diabetes, cerebrovascular disease, mental illness, hepatitis, tuberculosis, and no family history of any hereditary disease.

On admission, the patient had a body temperature of 36.3°C, a heart rate of 91 beats/min, a respiratory rate of 20 breaths/min, and a blood pressure of 162/85 mmHg. The neurological assessment revealed that the patient had cognitive dysfunction (poor memory and poor calculation ability). However, other categories of the neurological examination, including cranial nerves, motor system, reflexes, sensation, coordination, movement, gait, and signs of meningeal irritation, were normal.

The serum testing of routine blood tests, coagulation profile, liver function and kidney function tests, blood sugars, lipid profile, and serological testing for hepatitis B, syphilis, and HIV were negative. In addition, the results of anti-nuclear antibodies, antineutrophil cytoplasmic antibodies, rheumatoid factors, thyroid-stimulating hormone receptor antibodies, anti-thyroglobulin antibodies, and serum tumor markers, including carcinoembryonic antigen, squamous cell carcinoma antigen, cytokeratin-19 fragment, carbohydrate antigen 199, carbohydrate antigen 125, and neuron-specific enolase were normal. The patient had abnormal serum chloride levels of 95mmol/L. Further, the serum AE-related antibodies determined with both tissue-based and cell-based indirect immunofluorescence (IIF) assay in V-Medical Laboratory (Guangzhou, China), including anti-N-methyl-D-aspartate receptor (NMDAR), anti-leucine-rich glioma-inactivated 1 (LGI1), anti-contactin-associated protein-like 2 (CASPR2), anti-gamma-aminobutyric-acid B receptor (GABABR), anti-dipeptidyl-peptidase-like protein-6 (DPPX), and anti-glutamic acid decarboxylase 65 (GAD65) and paraneoplastic neurological syndrome (PNS)-related antibodies including anti-Hu, anti-Ri, anti-Yo, anti-Ma2, anti-CV2, and anti-amphiphysin, was remarkable only for positive anti-LGI1 antibody with a titer of 1:16 (normal <1:10). The CSF showed slight leukocytosis of 10 × 10^6^ / L with lymphocytic predominance. However, the pressure, color, turbidity, glucose levels, chloride levels, Gram staining, acid-fast staining, ink staining, metagenomic next-generation sequencing (mNGS), AE-related antibodies which were also determined with both tissue-based and cell-based IIF assay in V-Medical Laboratory (Guangzhou, China), and PNS-related antibodies of the CSF were normal. The patient had a positive RPR with a serum titer of 1:16 and a positive TPPA. Subsequently, TPPA and RPR in CSF were tested, and both results were positive.

**Figure 1:**
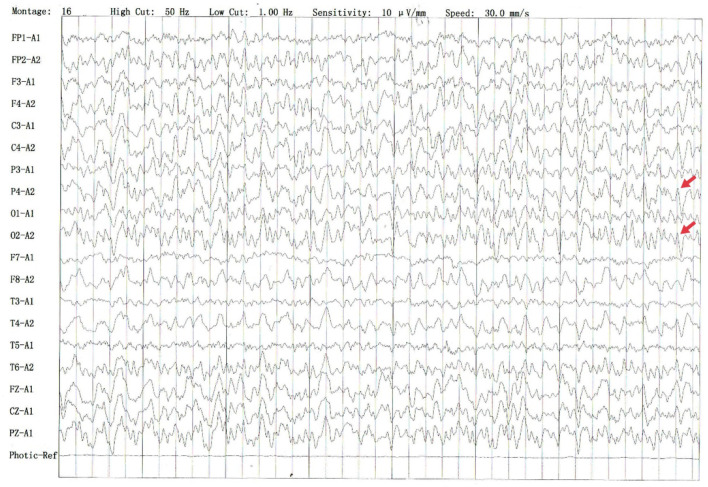
Several irregular slow waves with medium to high amplitudes were recorded in the right temporal leads, which spread to other leads and showed sharp waves (shown by the red arrow).

**Figure 2:**
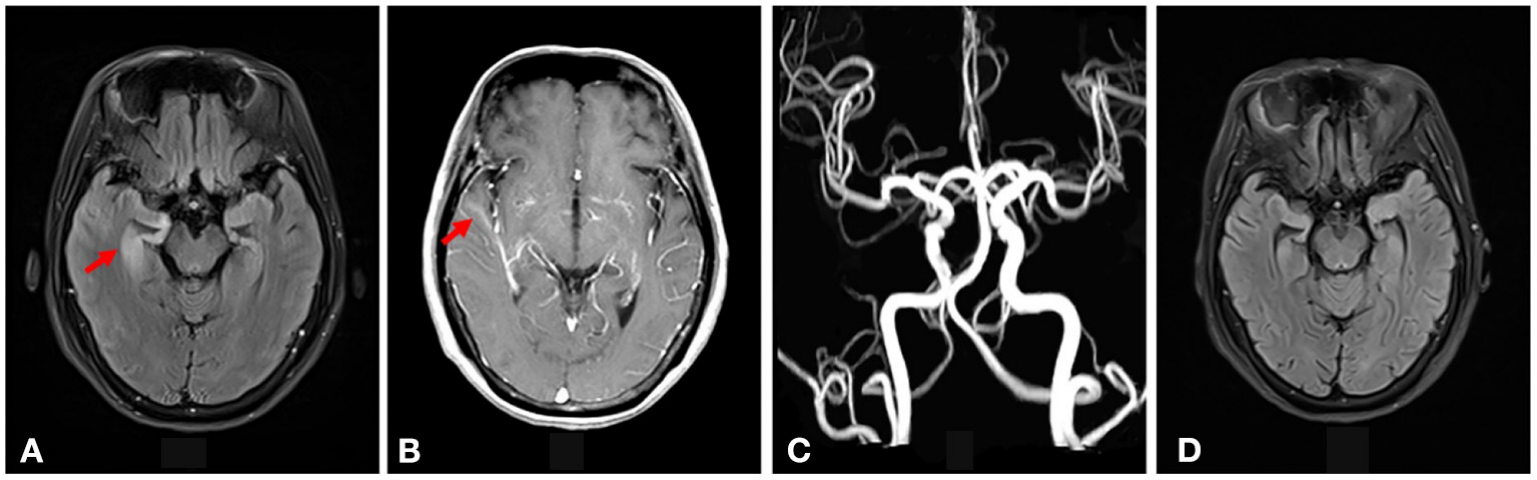
**(A)** Increased signal intensity was seen on the T2-weighted FLAIR imaging in the right medial temporal lobe (shown by the red arrow). **(B)** Gadolinium-enhanced MRI of the brain showed mild to moderate cord enhancement in the right temporal lobe (shown by the red arrow). **(C)** The brain MRA did not show vascular stenosis or vasculitis-like changes. **(D)** The increased signal intensity on T2-weighted FLAIR imaging in the right medial temporal lobe disappeared after syphilitic treatment.

The electroencephalography (EEG)showed irregular slow waves with medium to high amplitudes in the right temporal lobe, which spread to the other lobes and showed sharp waves (Figure 1). The brain MRI showed increased signals on T2-weighted and FLAIR imaging in the medial temporal lobe (Figure 2A). The gadolinium-enhanced MRI of the brain showed mild to moderate cord enhancement in the right temporal lobe (Figure 2B). Syphilis can cause multiple system damage. Therefore, magnetic resonance angiography (MRA) was carried out to investigate vascular stenosis or vasculitis-like changes. However, the results revealed no abnormalities (Figure 2C). Moreover, CT of the chest, echocardiography, abdominal ultrasound, urinary tract ultrasound, electromyography, and nerve conduction velocities of the limbs were normal.

**Figure 3:**
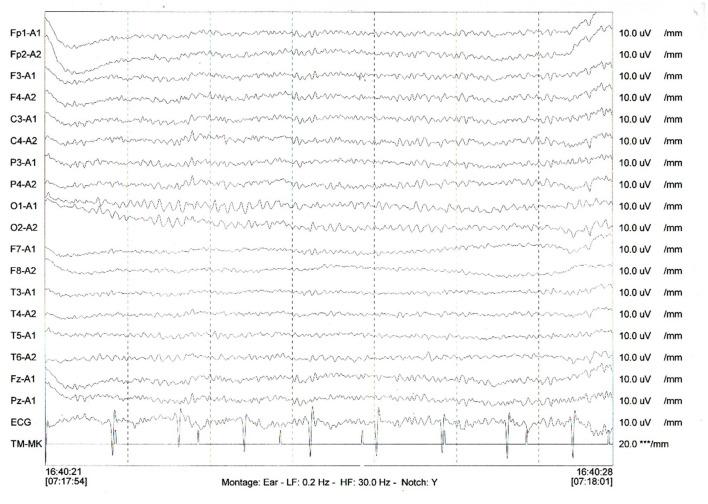
A repeat of the EEG showed no irregular slow waves and sharp waves emission. *** means uV, the unit of measurement of electromyography (EMG).

**Figure 4:**
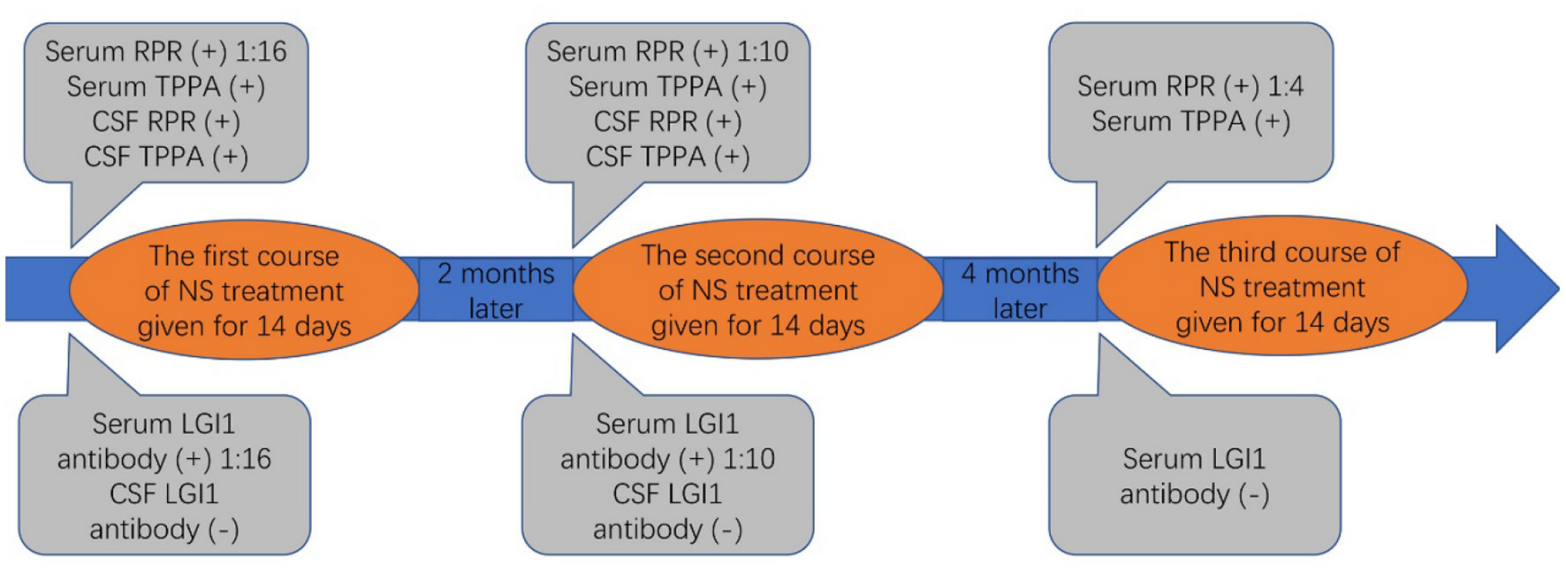
The changes in syphilis and LGI1 antibodies over the course of syphilitic treatment.

Therefore, uncertainty arose as to whether the patient had both anti-LGI1 encephalitis and NS or whether the LGI1 antibody and LE manifestations were due to the NS. The patient received intravenous penicillin sodium 3.5 million units every 4 h for 14 days to treat the NS. Furthermore, the patient was initiated on intravenous sodium valproate 400 mg two times daily and oral levetiracetam 500 mg two times daily. The drugs were then changed to oral sodium valproate 500 mg two times daily and oral levetiracetam 750 mg two times daily. Improvement was noted with no convulsions and normal cognitive function. Two months later, the serum TPPA was still positive. In addition, the serum RPR was still positive with a titer of 1:8, while the serum LGI1 antibody was positive with a titer of 1:10. Furthermore, a revaluation of the CSF showed that the CSF TPPA and RPR were positive. However, the other CSF tests remained negative. In addition, a repeat of the EEG showed no epileptiform wave emission (Figure 3). Moreover, a repeat of the brain MRI showed no abnormality (Figure 2D). A second course of intravenous penicillin G sodium 3.2 million units every 4 h for 14 days was started. After another four months, the serum TPPA was still positive, the serum RPR test was positive with a titer of 1:8, while the serum LGI1 antibody was negative. Changes in TPPA, RPR, and LGI1 antibodies during the syphilitic treatment are shown in Figure 4. A repeat of the EEG and brain MRI showed normal findings. The third course of intravenous penicillin G sodium 3.2 million units every four hours, was given for 14 days. In addition, oral doses of sodium valproate 500 mg two times daily and levetiracetam 750 mg two times daily were continued. The patient was then followed up for additional three months. The patient reported no further convulsive episodes. In addition, the memory and calculation ability were noted to be normal.

## Discussion

We describe an unusual case of NS mimicking LE which has rarely been reported in literature. Based on the clinical manifestations and features of brain MRI, this particular case is identified as a limbic encephalitis. Limbic encephalitis is an inflammatory process of the limbic structures, with polymorphic clinical features, caused by paraneoplastic and nonparaneoplastic conditions and infections. In this case, it was important to consider some relevant differential diagnoses including paraneoplastic LE, herpes simplex encephalitis (HSE), and anti-LGI1 encephalitis.

Paraneoplastic LE is a paraneoplastic syndrome associated with antineural antibodies produced by tumors against intracellular antigens. The classical clinical presentation is characterized by subacute cognitive deterioration, especially short-term memory loss, seizures, and psychosis, suggesting involvement of the limbic system (6). PNS-related antibodies include anti-Hu, anti-Ri, anti-Yo, anti-Ma2, anti-CV2, and anti-amphiphysin. In this case, the diagnosis of Paraneoplastic LE was excluded by exclusion of cancer and the negativity of PNS-related antibody in both serum and CSF.

Herpes simplex encephalitis mainly presents with a subacute progression of fever, hemicranial headache, behavioral abnormalities, focal seizure activity, and focal neurological deficits (7). Mesiotemporal T2-weighted hyperintensity with an asymmetrical pattern also can be found in HSE. The diagnosis of HSV encephalitis was excluded by the clinical presentation and the negativity of mNGS for herpes simplex in CSF.

Anti-LGI1 encephalitis commonly present with seizures especially FBDS and cognitive decline mainly including memory and behavioral disturbance (8). Ancillary testing features of anti-LGI1 encephalitis, include mild to moderate hyponatraemia, normal CSF test results, or a slightly increased cell count in CSF, unilateral or bilateral hyperintensities in the medial temporal lobes in brain MRI and focal slowing or epileptic discharges in EEG (9). According to the patient's clinical symptoms including FBDS-like manifestation and cognitive deficit, a slightly increased cell count in CSF, hyperintensity in the medial temporal lobes in brain MRI and serum positive LGI1, we initially considered diagnosis of anti-LGI1 encephalitis.

However, the serum was positive for TPPA and RPR, followed by positive TPPA and RPR in CSF. Therefore, there was uncertainty whether the patient had both anti-LGI1 encephalitis and NS or whether the NS induced the LE manifestations and the production of the LGI1 antibody. Treatment with penicillin G sodium according to the latest European guidelines(10), showed a significant improvement in the clinical symptoms, a decreased signal intensity on T2-weighted FLAIR imaging in the right medial temporal lobe, and a decrease in the LGI1 antibody titer. Therefore, it was assumed that the NS induced the LE manifestations and the production of the LGI1 antibody.

Neurosyphilis is an infection of the CNS caused by *Treponema pallidum*. It damages the meninges, blood vessels, or parenchyma of the brain and the spinal cord. The estimated incidence of NS is about 0.47 to 2.1 per 100,000 people (3, 11). Our patient's exclusive partner of more than 30 years tested seropositive for syphilis, and he admitted occasional sexual activity outside of this relationship. It is therefore possible that the patient contracted syphilis from her long-term partner in the course of sexual activity

The clinical manifestations of NS are related to the duration of the infection (12). Early-stage neurosyphilis usually is characterized by asymptomatic meningitis, but it can be symptomatic with headache, meningismus, cranial-nerve palsies, and blindness or deafness. Meningo-vascular syphilis is seen in early and late NS and involves the small and medium-sized arteries of the central nervous system leading to vasculitis, stroke, and several types of myelopathies. Late neurosyphilis occurs decades after the initial infection and is generally characterized by general paresis including progressive dementia, psychiatric syndromes, personality change, manic delusions, tremor, dysarthria, Argyll Robertson pupils in fewer than half of patients and tabes dorsalis including ataxic gait, prominent Romberg's sign, lightning pains in legs and trunk, greatly impaired deep and proprioceptive sensation, Charcot joints, Argyll Robertson pupils in most patients, paraparesis with leg areflexia, sphincter dysfunction (12). Currently, NS presents with atypical clinical forms due to incomplete doses of antibiotics for the treatment of other infections (4). In our case, the patient presented with gibberish speech, cognitive impairment, and seizures, characteristic of LE.

In the presented case, the serum and CSF RPR and TPPA tests were positive, suggesting a positive diagnosis for NS. NS is usually accompanied by CSF pleocytosis with 97% specificity and 95–100% sensitivity, and CSF elevated protein with less than 50% specificity and 90-95% sensitivity (12). A repeat white blood cell count in the CSF is used to determine the effectiveness of treatment. Re-treatment is recommended if the cells in the CSF do not decrease within six months of treatment (13). According to a previous study, there is no need to repeat treatment if the serum RPR titer is reduced four times or is negative(14). In this case, a slight increase in the white blood cells count was noted in the first CSF examination. Then, the patient's CSF white blood cell count decreased to normal and the serum titer of RPR decreased four times after treatment.

The common imaging manifestations of NS show abnormalities in the branches of the middle cerebral artery and basilar artery (15). Moreover, it has been reported that brain MRI in NS is often characterized by atrophy, white matter lesions, cerebral infarction and edema (4). So far, only a few cases have reported high signal intensity of the medial temporal lobe structure similar to LE on brain MRI (16–18).

The most prominent feature of this case is that both the first and second LGI1 antibody tests are positive, based on tissue-based and cell-based IIF assay, so that it is almost misdiagnosed as anti-LGI1 encephalitis. Ultimately, the patient's clinical symptoms improved significantly in the absence of glucocorticoids and gamma globulin therapy help us to rule out the diagnosis of LGI-1 encephalitis. In our view, NS may not only show LE manifestations but also cross-react with AE, leading to false-positive LGI1 antibodies. In our case, the effectiveness of antibiotic therapy suggests that NS may lead to a secondary immune response to LGI-1 antibody. It has been reported that the deterioration in neurological function after several weeks of treatment in HSE or its recurrence after viral clearance is related to the immune response associated with the production of NMDAR antibodies. Patients may present with deterioration in clinical manifestations, including abnormal mental status, cognitive dysfunction, and seizures similar to limbic encephalitis (19, 20). Therefore, we proposed that *Treponema pallidum* infection of the central nervous system may trigger an autoimmune response in the brain. Moreover, there is a need to investigate the presence of antibodies against synaptic receptors and intracellular antigens in patients with NS.

Based on this, we recommend that patients with LE, be tested for syphilis to avoid misdiagnosis in patients with atypical forms of NS and positive AE antibody in NS patients need to be viewed and interpreted carefully.

## Data Availability Statement

The raw data supporting the conclusions of this article will be made available by the authors, without undue reservation.

## Ethics Statement

Written informed consent was obtained from the individual(s) for the publication of any potentially identifiable images or data included in this article.

## Author Contributions

HL initiated the case report and drafted the manuscript. YZ consulted the relevant literature. WY were responsible for formulating the patient's treatment plan and revising the manuscript.

## Conflict of Interest

The authors declare that the research was conducted in the absence of any commercial or financial relationships that could be construed as a potential conflict of interest.

## Publisher's Note

All claims expressed in this article are solely those of the authors and do not necessarily represent those of their affiliated organizations, or those of the publisher, the editors and the reviewers. Any product that may be evaluated in this article, or claim that may be made by its manufacturer, is not guaranteed or endorsed by the publisher.
